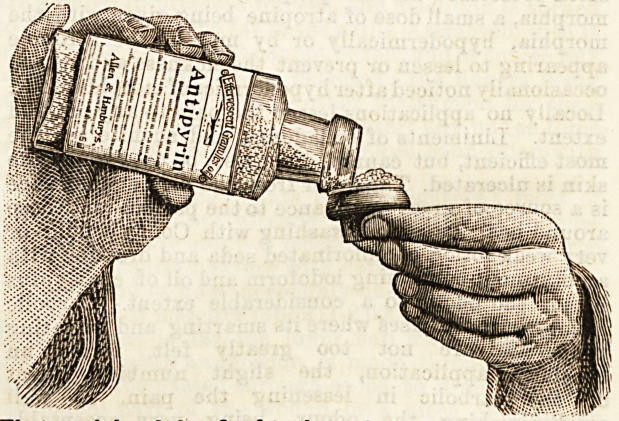# New Appliances and Things Medical

**Published:** 1893-07-22

**Authors:** 


### NEW APPLIANCES AND THINGS MEDICAL.

[All preparations, appliances, novelties, etc., of which a notice is desired, should be sent for the Editor, to care of The Manager, 428, Strand, London, W.C.]

### NEW SERIES OF GRANULAR EFFERVESCENT PREPARATIONS.

(ALLEN AND HANBURYS.)

We have received samples of the above for examination and notice. Coming from a house with such a well-established reputation as Allen and Hanburys' we expect the highest results from analysis for purity and exactness of dosage. Such results we obtained. But in addition to these common virtues, if we may so call them, of chemical manufacture, there are many interesting and valuable qualities attached to this new series of effervescent preparations.

First, each bottle has fixed to the cork, in such a manner that it cannot be lost, a boxwood measure. This, when full, contains the correct minimum dose. In itself this is no small advantage, as taking a dose of ordinary effervescing saline by shaking a quantity into a tumbler and then adding water is but a rough-and-ready method which, if applied to an effervescent containing a more powerful drug, such as antipyrin, might lead to serious mishaps. With regard to the particular preparations under review the following points are to be noted: The granules are all small and of regular size, so giving an even dose and doing away with a mass of large granules at the top of the bottle and small ones at the bottom, and thus removing the chance of "heel-tap" over dosage, and the unpleasantness of a mouthful of large undissolved lumps of effervescent often met with in the old-fashioned granular preparations. In thus favourably noticing these very elegant and convenient preparations, we pay a well-deserved compliment to a firm which has always been well to the fore in the English drug trade, and which has a reputation for skill and enterprise second to none.

## Figures and Tables

**Figure f1:**